# The speculative turn in IVF: egg freezing and the financialization of fertility

**DOI:** 10.1080/14636778.2019.1709430

**Published:** 2020-03-18

**Authors:** Lucy van de Wiel

**Affiliations:** Sociology, University of Cambridge, Cambridge, UK

**Keywords:** egg freezing, fertility, IVF, financialization, cryopreservation, reproductive technologies

## Abstract

Although IVF and egg freezing have received much scholarly attention, the pivotal role of financialization in the fertility (preservation) sector remains understudied. This article discusses how processes of financialization have instigated a step-change in the organization of contemporary US IVF and why egg freezing is at the heart of a wider consolidating trend in the sector. The financialization of fertility, in this context, references the financial investments in a future in which ever more women freeze their eggs, the role of capital markets in establishing new clinical and commercial infrastructures through which egg freezing becomes accessible and the role of financial products in shaping both the stories and the streamlining of fertility treatments. Together, these developments signal a shift from reproduction to fertility in IVF, in which treatment is not aimed at having a child at present, but rather at the proactive management of a more speculative fertility throughout the life course.

## Introduction

Contemporary IVF is undergoing a speculative turn, which is characterized by an increasing number of tests and treatments that are future-oriented, risk-focused and speculative in nature. Beyond a treatment for current experiences of infertility, IVF is increasingly oriented towards the pre-emptive and proactive treatment of future infertility. This proactive approach is reflected in the growing popularity of cryo-preservation technologies – particularly oocyte cryopreservation (OC), or egg freezing – both in existing fertility clinics and in new specialized start-ups. In the US, fertility companies provide and heavily market egg freezing as a widely-indicated means of counteracting age-related fertility decline. Thus changing the indication for fertility treatment, predictive technologies for fertility testing have also become an integral part of a new ethos of proactive fertility management. Major IVF clinics offer fertility check-ups – so-called “fertility MOTs” – and a growing number of start-ups specialize in data-driven testing innovations, which use reproductive health data and predictive analytics to offer personalized estimations of future reproductive chances. Investments in these preservation and prediction technologies reflect how a speculative orientation to the futurity of fertility is increasingly central to contemporary IVF practices.

In part, this speculative turn follows the emergence of new oocyte vitrification technologies which significantly improved the prospects for female fertility preservation (Kuwayama *et al*. 2005). Especially after the ASRM’s 2012 declaration that egg freezing was no longer considered “experimental” – and in spite of the Society’s less widely-quoted reservations about OC’s use to circumvent age-related infertility – egg freezing has rapidly gained in popularity and is now on offer in 97% of US IVF clinics (ASRM 2012; CDC 2018, 22). Uniquely, egg freezing is both an infertility treatment for the fertile and a fertility treatment for the infertile. Younger, fertile women are freezing their eggs in preparation for future infertility, while frozen eggs enable the possibility of conception after the onset of age-related infertility. Through this double movement, categories of fertility, infertility and what we may call “postfertility” are mobilized in new ways. This article addresses how processes of financialization are at the heart of this step-change in the “proactive” recognition and treatment of future infertility in contemporary IVF.

The speculative turn in assisted reproduction is also characterized by speculative investments in these technologies by investors, entrepreneurs, employers and patients alike. Although the relation between reproduction, cellular life and capital has been extensively theorized through concepts of biocapital, biovalue and bioeconomies (see Helmreich (2008) for an overview), the shifting power dynamics in the fertility sector resulting from large equity investments, increasing consolidation and the institutionalization of financial instruments for IVF payment have received relatively little attention. While scholarship on egg freezing has addressed the neoliberal rationalities underlying “fertility preservation,” this paper argues that processes of financialization are equally crucial to understand the rising popularity of egg freezing. It does so by focusing on the role of financialization in establishing the infrastructures through which OC may be accessed and considers, by extension, how the recent emergence of egg freezing is re-organizing reproductive healthcare more broadly.

Focusing on egg freezing specifically, this article seeks to characterize the *financialization of fertility* and its crucial role in shaping the contemporary IVF institutional landscape. I first develop a conceptual framework in dialogue with the literature on financialization, biocapital and cellular life. Focusing on fertility, I subsequently describe the role of equity investments in the emergence of egg freezing start-ups and the concomitant expansion and consolidation of fertility services.^1^ I then describe how financial products such as subscription plans and insurance for egg freezing establish a dynamic between investment and indebtedness through which ongoing fertility is enacted and constituted. Drawing on Melinda Cooper’s (2008) and Sarah Blacker’s (2014) work on financialization in regenerative and genomic medicine, I discuss how both the material conditions and the underlying logics of financialization function as enabling conditions and interpretative frames for a reinvented, speculative and precarious notion of fertility and its futurities.

The financial investments and broader commercial and clinical infrastructures constructed around the promise of fertility preservation, of course, do not determine specific experiences of egg freezing, but they nonetheless do play a key role in constructing the material realities that organize OC practices, thereby mainstreaming egg freezing across larger groups of potential patients, integrating egg freezing into future treatment plans and rationalizing OC through new treatment logics that are changing what it means to be fertile in the twenty-first century.

### Financialization, fertility and cryopreservation

Dominant discourses of egg freezing – particularly so in the US context – align neatly with neoliberal rationalities by appealing to ideas about “self-responsibilization” for the ongoingness of fertility and maximization of one’s “human capital” through the enhancement of future reproductive potential (Brown 2015). The growing body of scholarship on egg freezing has addressed OC in relation to the neoliberal subject, who bears a heightened responsibility for reproductive ageing, and popular risk-focused discourses on fertility characterized by an “implicit injunction to stay informed [and] to live the future in the present body” (Van de Wiel 2015, 123). Carroll and Krolokke analyze egg freezing as an enactment of “responsible” reproductive citizenship that “anticipates coupledom” and genetic relatedness (2018). Rottenberg likewise reads egg freezing as symptomatic of a middle-class neoliberal governmentality based on smart self-investments for enhanced returns in the future, while Emily Jackson highlights the possibility of blame and retrospective regret as the flipside of this responsibilization of one’s future fertility (Rottenberg 2016; Jackson 2017).

In this article, I analyze how the widely-observed neoliberal rationality of OC is situated in the context of regimes of financialized capitalism that are instrumental in creating the emergent clinical and commercial infrastructures through which egg freezing has become accessible in the first place. The financial systems and logics underlying the organization of the US fertility industry are key to understanding the current popularity of egg freezing and the neoliberal governmentalities it exemplifies.

Current egg freezing practices are positioned against the backdrop of what Fraser calls “regimes of globalizing, financialized capitalism” (2015, 167).^2^ In contrast to a postwar state-managed capitalism, financialized capitalism “authorizes finance capital to discipline states and publics in the immediate interests of private investors” (2018, 75). Fraser argues that financialized capitalism remakes the constitutive institutional separation of reproduction and production through a move from the Fordist family wage to the ideal of the two-earner family. This shift is accompanied by the “steep rise in the number of hours of paid work now required to support a household,” which effectively entails an obligation to “shift time and energies once devoted to reproduction to ‘productive’ (i.e. paid) work.” In light of these developments, Fraser contends egg freezing is symptomatic of a social organization which requires “shoehorn[ing] social reproduction responsibilities into the interstices and crevices of lives that capital insists must be dedicated first and foremost to accumulation” (2018, 87). In this process, egg freezing not only functions as a resolution to this scarcity dynamic between production and reproduction, but also brings fertility itself into the realm of accumulation.

Of course, from its very inception, fertility treatment has been closely aligned with capital accumulation and privatized healthcare. In the UK, where the first IVF baby was born and the first IVF clinic was founded in 1980 by the clinicians responsible for Louise Brown’s birth, the emergence of this new medical sector coincided with Margaret Thatcher’s rise to power. Marilyn Strathern has described the “enterprising up” of IVF in this context and Sarah Franklin has analyzed IVF in relation to the “enterprise culture” of Thatcherism (Strathern 1990; Franklin 1997). Gay Becker documented the embeddedness of IVF experiences in the ethos of the American Dream (Becker 2001, 39; Franklin 2013, 240). The neoliberal responsibilization of future fertility with OC emerges in the wake of these histories of IVF.

Yet egg freezing is also quintessentially a reproductive technology of the contemporary moment, in which a shift towards financialization in the fertility sector – particularly the largely private US sector – meets a speculative turn in IVF enabled by (cryo)preservation and prediction technologies. Financialization here includes “changes in management ideology that increasingly orient firms to financial markets (i.e. ‘shareholder value’),” “the growing influence of financial products, [and] the extension of debts in underserved communities” (Krippner, Lemoine, and Ravelli 2017). To understand the phenomenon of egg freezing, then, we need to not only focus on clinicians and patients, but also on the firms and financiers that shape this part of the reproductive bioeconomy. This requires addressing not only the sale of commodities (e.g. revenues for goods and services), but also the financial value ascribed to egg freezing by the capital markets and their investments in fertility companies (Birch 2017, 472). What is at stake in this focus on financialization in the fertility sector is not so much the fact of commercialization, but rather the shift in power relations and the reconceptualization of female fertility in the face of the changing financial dynamics that govern the industry and *its* viability.

A small body of scholarship in critical political economy analyses the relation between financialization and the biotechnology sector (Hogarth 2017, 253). This work draws attention to the way in which value is assigned on the basis of a speculative estimation of future profit when institutions operate according to a financialized logic (Blacker 2014, 127). The promise of future value – whether produced through future profit margins or future acquisitions and exits – can attract investment capital. In healthcare, financialization has resulted in the expansion of markets, the development of private insurance markets alongside public programs, a managerial focus on shareholder value and speculative “expansions, mergers, and acquisitions aimed at profit maximization and consolidating market advantage” (Mulligan 2016, 39).

In *Life as Surplus*, Melinda Cooper theorizes financialization and bioeconomies in relation to stem cell technologies. Cooper describes how the encounter with the limits of industrial production in the 1970s recession opened up “new forms of production and accumulation” through financial investment in biotechnologies that relocated accumulation “beyond the limits of industrial production – in the new spaces opened up by molecular biology” (Cooper 2008, 22). She argues that the flooding of venture capital into biotech companies was a clear sign of how speculation had become “the driving force behind unprecedented levels of innovation, allowing whole industries to be financed on the mere hope of future profits”-particularly so in the biotech sector, where cellular life “bec[a]me intimately infused with the virtual temporality of speculation” (Cooper 2008, 96). In a similar vein, Philip Mirowski characterizes the “biotech firm” as a “financial artifact” because these firms “are not primarily configured as technoscientific organizations – that is, as producers of technoscience or technoscientific products – but, instead as financial organizations” because most biotechs never produce a drug or final product (qtd. in Birch 2017, 464–65). Instead they reflect the “promissory character of contemporary capitalism” and the way in which “the promissory is transformed into the real and the role of VCs, market analysts and public exchanges in this process” (Martin qtd. in Hogarth 2017, 252–53). Yet while the financialized bio-technology sector “has not lived up to expectations” either financially or technoscientifically (Birch 2017, 471), the fertility industry is a biomedical sector that *does* show both financial and clinical returns, through which trends of financialization and its relation to cellular life may be analyzed differently.

Theorizing the relation between the biological and the financial, Cooper argues that the growth potential materialized in the stem cells’ *generativity* matches the growth drive of financialized capitalism. Following this work, this article explores how cellular *cryopreservability* likewise “becomes annexed within capitalist processes of accumulation” (2008, 19). It draws attention to the growing importance of fertility – to be distinguished from reproduction – within the accumulation strategies of the US IVF sector, focusing particularly on the relation between the accumulation of reproductive time and the accumulation of capital through OC. This article thus explores the relation between cryopreservation and financialization in the new forms of indebtedness, financing and investment co-emerging with contemporary egg freezing practices.

### Equity for cryo-eggs

**Figure 1:**
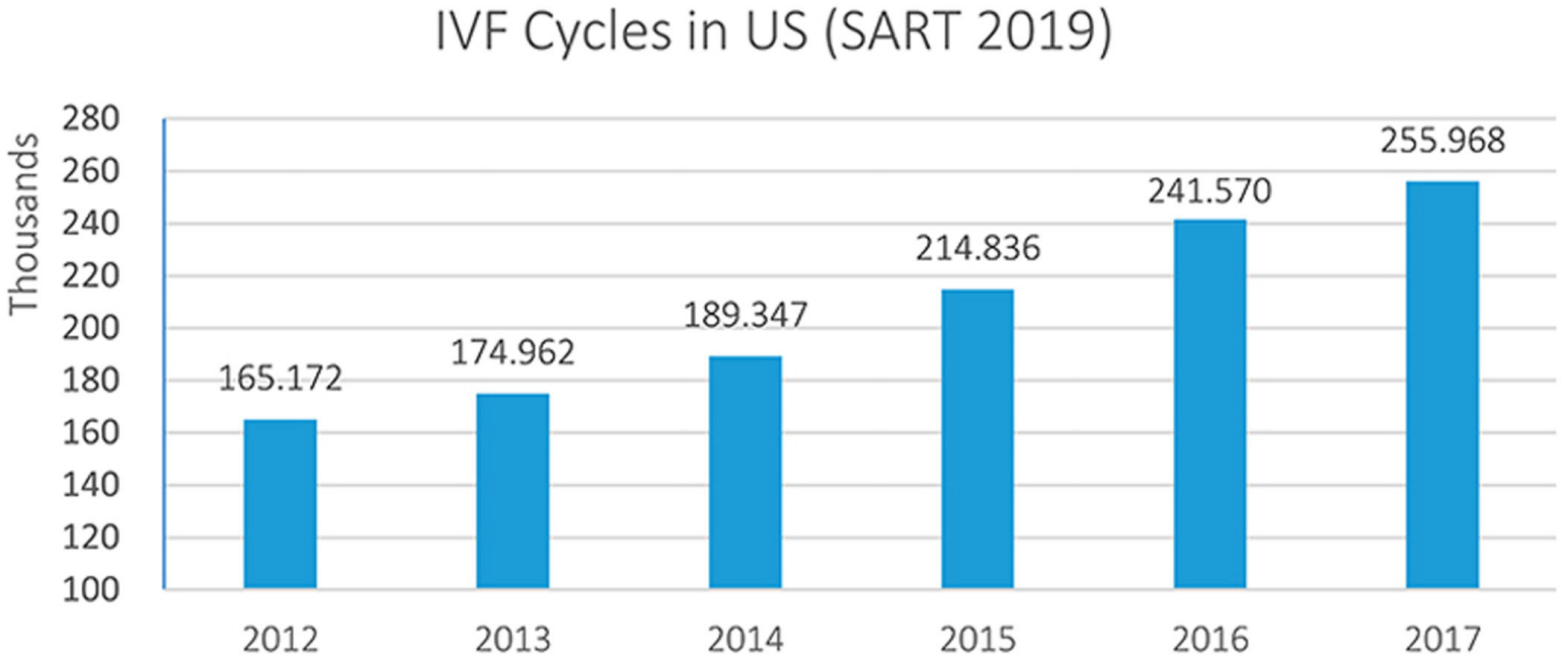
IVF Cycles in US (SART 2019).

**Figure 2:**
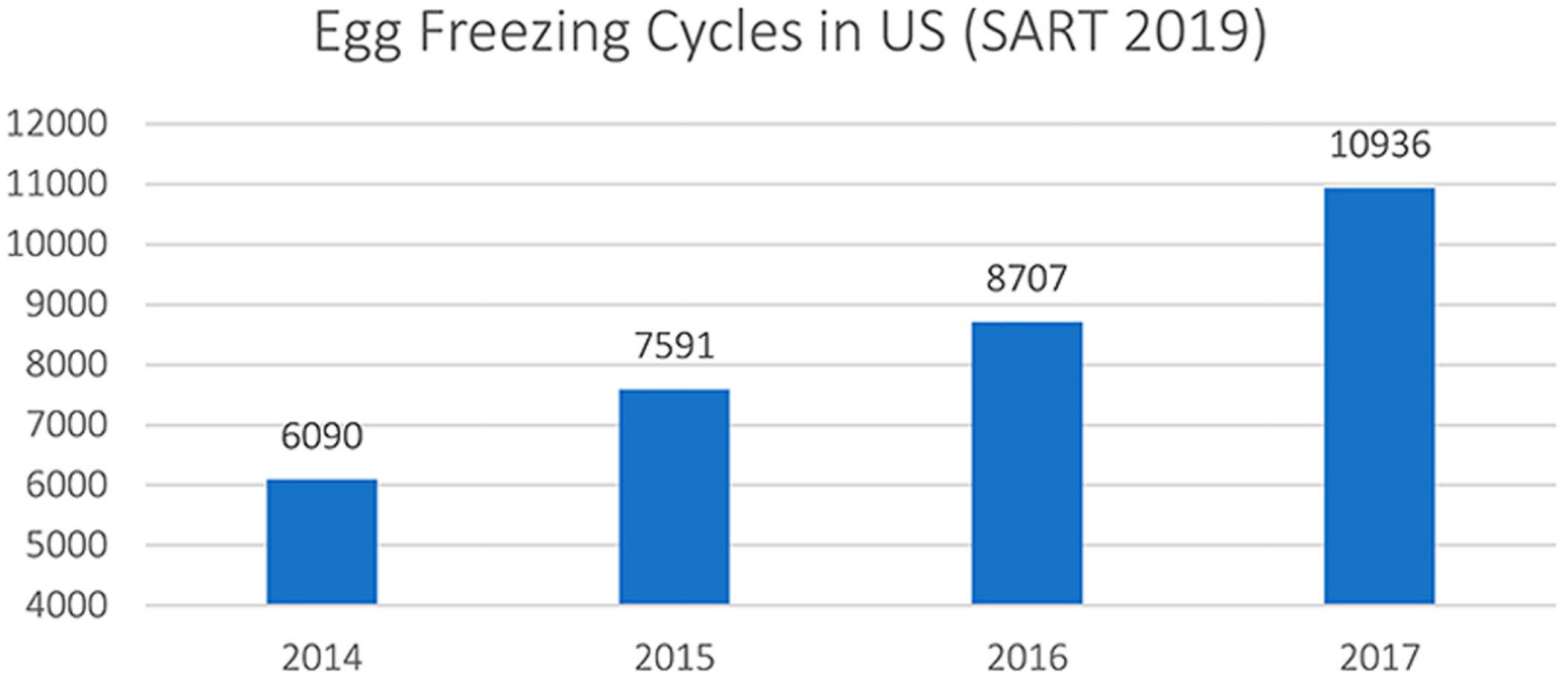
Egg Freezing Cycles in US (SART 2019).

The increasing popularity of egg freezing is situated in a global fertility sector that has experienced consistent growth and is projected to continue expanding to an estimated $36 billion by 2026 at an annual growth rate of over 10% (GVR 2019).^3^ In keeping with this trend, the total number of IVF cycles in the US has steadily grown every consecutive year (see Figures 1 and 2). Although egg freezing only accounts for a small percentage of US IVF cycles – less than 4% are performed for oocyte banking even though the procedure is on offer in 97% of clinics – this technology has received widespread attention in popular media and academic scholarship (CDC 2018; SART 2019). In spite of these small – albeit rapidly growing – numbers of women freezing their eggs, the promise of cryopreserving female fertility has also attracted investors’ interest. Since 2016, millions of dollars of private equity (PE) and venture capital (VC) have been invested in egg freezing businesses, which materialize the promise of egg freezing as a growth technology that may be targeted at a wide group of younger, fertile women, who may or may not want to have children in the future – a far greater segment of the population than those currently accessing IVF.

Buoyed by VC and PE investments, egg freezing-focused start-ups are emerging rapidly and are changing the landscape of US IVF. Prelude Fertility, for example, is one major new player focused on egg freezing, which was founded in 2016 with the aid of $200 million equity investment. Extend Fertility has operated for a decade through its network of IVF clinics and, in 2016, opened the world’s first egg-freezing only clinic in New York. This business is backed by private equity from North Peak Capital and received a further $15M in 2019 from Regal Healthcare Capital Partners. Kindbody was founded in 2018 with $6.3M seed funding and describes itself as “the future of women’s health, fertility and wellness.” It brings egg freezing to the streets of urban centers with yellow “fertility vans,” or “boutique mobile locations,” which offer information on egg freezing and on-site fertility testing (Kindbody 2019). In 2017, embryologist Colleen Wagner Coughlin founded Ova Egg Freezing in Chicago as part of the four business entities of which she is the sole owner: Gamete Resources, Ova Institute, Cryovault and Egg Bank Foundation (Ditkowsky 2018). Ova Egg Freezing is a member of the California Cryobank Donor Egg Bank USA Network – a major cryopreservation company that combines sperm, egg and cord blood banking after a massive merger and acquisition deal worth an estimated $1 billion by San Francisco-based private equity firm GI Partners (Ova 2018; Ditkowsky 2018).

Beyond clinical services, some of the major new egg freezing start-ups offer financial products. The most high-profile company is Progyny, which sells fertility benefits covering egg freezing to employers. Fertility insurance was at the heart of an international media hype when Apple and Facebook began offering egg freezing benefits in 2014; since then, a growing number of Fortune 500 companies have adopted fertility insurance packages. Progyny secured almost $100 million in equity to grow its corporate fertility benefit business, a process which has been aided by a strategic alliance with Mercer, the world’s largest HR company (Lee 2016; Crunchbase 2019b).^4^ Although growth figures are not public, in 2018 Progyny was named #3 on Crain’s Fast 50 List, which records the fastest-growing companies in New York based on growth in revenue in 2014–2017; listed companies have an average three-year growth rate of 2082%, so as the third Progyny should be well above that (Yang 2018). Carrot Fertility is a smaller company that secured $3.7 seed funding to provide fertility health benefits and a digital platform to employers. Future Family received $114M in venture capital and debt financing to offer loans and subscription plans for egg freezing and other fertility treatments (Crunchbase 2019a). Symptomatic of the financialization of fertility, the funding attracted for these companies highlight the significance of financial products in the mainstreaming of US egg freezing.

Collectively, these new egg freezing companies have a widespread reach; they manage relations with nation-wide networks of fertility clinics, manage influential online platforms and its innovations receive widespread media and academic commentary. The emergence of these for-profit egg freezing ventures is situated in the context of the US capital market, which is much larger than its European rivals; US start-ups therefore enjoy some relative advantage when looking for capital (Hogarth and Salter 2010). Reflecting the prominence of financialization in the wider US economy, there has been a steady growth in US equity investments over the last years; 2018 was an all-time high with investments totaling $130.39 billion, a 50% increase from the previous year, and breaking the record set during the dot-com bubble in 2000. The San Francisco Bay area, including Silicon Valley, was home to 61% of all capital invested into US companies, displaying “by far the most extreme regional concentration ever seen,” and followed by the New York area (Dow Jones 2019, 2–3). Progyny’s investors, Future Family, Carrot Fertility, and of course California Cryobank are all based in the Bay Area, with New York being home to Prelude’s main investor Lee Equity and the headquarters of Progyny and Kindbody.

Rather than simply providing the means for their emergence, the significant investment capital poured into egg freezing companies propels a much broader transformation in assisted reproduction. Birch and Tyfield describe the biotechnology sector as “underpinned by a rentier regime in which financial asset values are more important than revenues from the sale of biotechnology commodities” (2013, 322). In other words, key is not primarily the amount of revenue the company generates, but the (speculative) value of the company itself, based on its potential for future growth. The significant capital investment in egg freezing companies points to a valuation of their potential for future growth. These financial investments thus at once enable the current emergence of new egg freezing enterprises, signal the valuation of the promissory value of OC and materialize the speculation of further growth of this practice in the foreseeable future.

The private equity investments in egg freezing companies, then, point not simply to the capital market’s interest in the profit that may be generated from (more) women freezing their eggs. Rather, I will argue it reflects a more ambitious vision that positions cryopreservation at the heart of a step-change from reproduction to fertility in contemporary IVF. As Stuart Hogarth has argued in this journal, the ongoing growth of US private equity has led to a new model of business development, which relies on securing equity investment by presenting not simply a convincing business model, but a compelling vision for creating value that is by necessity futural and speculative in nature – and which aligns with the investment culture organizing relevant capital markets. For example, Hogarth argues that the investment culture of Silicon Valley is organized by the ideal of “disruptive innovation,” which is characterized by “a compelling vision of socially beneficial market transformation communicated by a passionate CEO, a belief in the transformative power of information technology” and “the ambition for global growth and market dominance” (2017, 256–58). By zooming in on the case of Prelude Fertility, we can consider the discursive, financial and infrastructural dimensions of equity-backed egg freezing enterprises and the “disruptive” visions of speculative fertility that propel them.

### Prelude and speculative fertility

The largest recent OC investment was $200 million committed to Prelude Fertility, an ambitious fertility company founded in 2016 that primarily focuses on egg freezing. Its CEO, Martin Varsavsky, is a serial entrepreneur specialized in real estate and cloud computing. FON, self-reportedly the world’s largest Wi-Fi network with 21 million hotspots across the globe, was one of his enterprises; Prelude Fertility is his seventh, and his first time branching out to the fertility sector. Varsavsky partnered with Lee Equity to acquire RBA IVF clinics and My Egg Bank, one of the largest US egg banks, to form Prelude Fertility (Dorbian 2016). With enough equity funding to acquire numerous existing IVF clinics, Prelude became the second largest US fertility company in its first year. Although 86% of US IVF clinics perform less than 1000 cycles annually, Prelude did over 10.000:

1. IntegraMed Fertility (including Shady Grove Fertility): 38,071 cycles.
2. Prelude Fertility: 10,740 cycles.
3. RMANJ: 8,474 cycles. (Dresner Partners 2018)

RMANJ is almost two decades old and Shady Grove Fertility was founded started 25 years ago by three physicians in a small Maryland office who claim never to have had “a master plan for SGF to expand as it has” (Cunningham 2017). Although Prelude’s cycles represent the activity of existing, acquired IVF clinics, this company reflects the “disruptive” effects of private equity and an experienced entrepreneur who is an outsider to IVF, but certainly does have a master plan for fertility.

Exemplifying Hogarth’s characteristic of a “charismatic CEO with a vision,” Varsavsky’s plan revolves around cryopreservation as a means to mainstream infertility treatment. Marketing Prelude as a “fertility company” rather than an infertility clinic, Varsavsky presents the so-called “Prelude Method,” a treatment package that combines cryopreservation, IVF and embryo genetic screening, as “a complementary strategy to starting a family by having sex”:

As opposed to people who solely rely on sex to make babies, people who rely on both sex and Prelude have a much greater chance of achieving their parental goals of having healthy babies when they are ready. Prelude uses the technology available to infertile people, on fertile people. At Prelude we believe that something as important as having […] a healthy baby, should not be left to chance. (Varsavsky 2016)

Blurring distinctions between those who do and don’t need fertility treatment, the Prelude Method exemplifies postfertility and echoes Sunder Rajan’s description of a parallel phenomenon in postgenomics: “a reconfiguration of subject categories away from normality and pathology and toward variability and risk, thereby placing *every* individual within a probability calculus as a potential target for therapeutic intervention” (2006, 167). Similarly, the Prelude Method both expands the target population of IVF to fertile people and expands the IVF cycle to include embryo screening technologies, thereby providing two axes of growth. Moreover, unlike the potential birth of a baby after an IVF cycle, egg freezing does not have an equally clear endpoint for marking reproductive success. The potential for repeat cycles to accumulate more cryo-eggs for further fertility assurance presents another rationality for growth.

Varsavsky’s vision of fertility care reflects a belief in the transformative power of technology – another characteristic of disruptive innovation (Hogarth 2017, 258). The belief that reproductive technologies can ensure that everyone can “have a healthy baby when [they] are ready” aligns with the vision that drove 23andme, the influential genetic testing company that started out with a similar amount of capital investment (Prelude Fertility 2017a). Its founder Anna Wojcicki said: “my goal isn’t to just minimize the chance of getting sick. I want to live a healthy life at 100” (Hogarth 2017, 259). The vision of healthy life extension by disrupting healthcare with genetic testing matches a vision of healthy reproductive life extension by disrupting fertility with (cryo)preservation and predictive technologies.

This approach to fertility extension later in life is matched with a model of proactive, technologized fertility risk management earlier in life. The notion that young women should be “proactive” in managing their fertility in the face of the progressive loss of their embodied eggs is at the core of several egg freezing companies’ missions. Prelude describes itself as “a comprehensive fertility company with a focus on providing proactive fertility care,” as reflected in its slogan: “It’s time to take charge of your fertility” (2017). Likewise, Extend Fertility presents itself as “the first service in the country to focus exclusively on women who want to proactively preserve their fertility options” and Progyny as the “leading digital healthcare company combining data and science to provide the first end-to-end, proactive fertility solution for employers” (Extend Fertility 2017; Bartasi 2016). The emphasis on proactive fertility care suggests a contradistinction with the existing – by implication reactive – model of IVF, in which people access treatment when they experience infertility or other barriers to reproduction. Instead, proactive fertility care requires active, technologized management earlier in life to “preserve options” and maintain the possibility of having (biogenetically-related) children later on.

Here speculative investment in the eggs’ freezability aligns with the promissory value of the future return – for patients, companies and investors alike. As promise becomes the “one fundamental of post-Fordist production,” which functions as a means to “anticipate and escape the possible ‘limit’ to its growth long before it has even actualized,” cryopreservation enables the temporal manipulation of cellular life to meet the speculative, futural orientation of a “finance-dominated regime of accumulation” (Cooper 2008, 23–24). A variation of the “double reproductive value” of stem cells (Franklin 2006), the freezable eggs here hold a double speculative value through the cryo-enabled promise of both a future financial return and a future return of fertility.

Varsavsky’s vision for the potential future growth of his fertility company – and the concomitant financial value for investors – is thus coupled with a reconceptualization of fertility that facilitates this future growth. Overcoming the “limits to production” inherent in a “reactive” model of IVF that relies only on the treatment of infertile people, the possibility of pre-emptively treating future infertility through cryopreservation broadens the target group, while the risk-avoidant Prelude Method allows for an expansion of each IVF cycle with additional genetic screening technologies.

It is this vision of a widely-indicated, extendable fertility that held the promise of future growth for Lee Equity, Prelude’s investors. Meeting the “ambition for market dominance” characteristic of disruptive innovation (Hogarth 2017), Prelude used their equity investment to acquire a nationwide network of 31 IVF clinics sprawling the US (Dorbian 2017; Ho 2017; Beltran 2018). While the US fertility industry is highly fragmented – 75% of clinics account for less than 0.24% of total cycles – egg freezing companies are establishing nationwide networks for fertility preservation. Whether through acquisitions (Prelude), strategic alliances with clinics (Progyny) or combining brick-and-mortar with mobile clinics (Kindbody), each of these companies have a broad geographical reach. The business case for investing in speculative fertility thus directly affects the landscape of US IVF as the equity investments enable network formations that position egg freezing companies as parent or umbrella organizations.

In order to reach a new group of potential patients, consolidated OC companies can also centralize marketing budgets to reframe IVF as a tool for comprehensive, proactive fertility management. A case in point, Prelude’s expansion through the acquisition of a growing number of clinics allows for the concentration of marketing efforts – and this is exactly what the investors had in mind. Lee Equity were interested in the growth potential of IVF, given the rising age of first-time mothers and the legalization of same-sex marriage. Yet they also recognized fertility awareness as a means to broaden demand for IVF. Collins Ward, a partner at Lee Equity, says that the “biggest surprise” he encountered in the fertility industry is the “low awareness of fertility services.” So the investment in Prelude was coupled with the “significant costs” of a big marketing push intended to, in Ward’s words, “speak to younger patients and younger Americans who live in social and digital media.” It is this drive to “increase awreness” that bears the promise of “a sizable upside in years to come” by proactively appealing to a new group of potential patients, who are themselves encouraged to be proactive about fertility (qtd. in Robbins 2017). Now comprising a nation-wide network, Prelude’s mission “to educate a generation of women of childbearing age about their fertility” and its “commitment to improving fertility awareness, and providing a proactive approach to family building” has a widespread reach (Prelude Fertility 2017b).

Prelude’s online platform reframes fertility in line with the company’s vision of mainstreaming proactive fertility management. It shows beautiful yet relatable young adults against a splash of stylish colors. Confident smiles are enframed with statements such as

Find that right person. Focus on your career. Finish your education. The age of your eggs (not you) is the number one cause of infertility. (Prelude Fertility 2017a)

Prelude’s website contrasts with the visuals of babies that dominate the majority of IVF websites (Hawkins 2013). Although other reproductive technologies are also on offer, the homepage prominently features egg freezing with a carrousel of quotes:

Stop the hands of the biological clock with Prelude

It used to be that women had few options, but not anymore

In keeping with these statements, Prelude’s slogan of “Options Preserved” echoes a vision that, as Strathern reminds us, has been there since the conception of IVF, when the idea that the “child ought to exist by choice” was embedded in the wider matrix of the prevalent “enterprise culture:”

NRTs are presented as opening up reproductive options, the vision of a biology under control, of families free to find their own form. (Strathern 1990, 3–4)

Yet here the focus lies less on the option of having a child and more on the continuation of fertility – as a precondition for achieving relationship, career and reproductive goals. In the absence of (the desire for) a child, fertility instead refers to a state of having options and the particular relation to futurity that implies.

Prelude’s invitation to “preserve your options” is counterbalanced by downward graphs that signal embodied egg loss. This framing of embodied fertility as ever in decline reflects a “capitalist promise [that] is counterbalanced by willful deprivation, its plenitude of possible futures counteractualized as an impoverished, devastated present, always poised on the verge of depletion” (Cooper 2008, 20). While the time of the body and the scarcity of eggs are construed as constraints to “having options,” the cellular temporal manipulation of cryopreservation affords their continuation.

Beyond marketing, the fertility companies’ online platforms are also key instruments in managing widespread networks. These platforms connect participating network members and take on functions previously covered by the clinic. Ongoing medical and emotional support is offered through concierge services and wellness apps (Progyny, Future Family), which provide a centralized discursive framing of the “entire fertility journey.” Kindbody takes this one step further through its patient portal, which provides the foundation for “building a centralized data platform, allowing for standardized decision-making, and building predictive protocols to define and scale best practices” (Kindbody 2018b). The equity-based egg freezing companies thus affect IVF’s broader infrastructure through acquisitions and network formation, the centralization of marketing and patient support through online platforms and the adoption of cloud-based services that enable standardization across the network.

### Financing fertility

As egg freezing infrastructures are thus expanding through financial investments, the resulting high stakes in increasing the number of women who freeze their eggs coincides with a shift towards interpellating younger potential patients to freeze now to take advantage of their “peak fertility.” The appeal to younger people, who typically have less access to the significant sums needed for egg freezing, is matched with financial products offered to broaden access to OC treatment, which represents another dimension of the financialization of fertility. Prelude, Extend Fertility and Future Family present subscription plans for egg freezing with fixed payments of $99-$300/month, while Progyny and Carrot Fertility offer egg freezing insurance to employers. This section discusses the major financial instruments adopted in the efforts to mainstream egg freezing and considers how they set up a dynamic of indebtedness and investment as part of contemporary cryopreservation practices.

Reflecting the trend towards promoting earlier freezing, Kindbody spreads the word about egg freezing and fertility decline by driving its fertility van through the streets of urban centers and offering passersby free fertility education and fertility tests. In the pastel-coloured yellow van, printed statements in photo frames convey the rationale behind earlier freezing:

You will never be as fertile as you are today.

Coupled with the “facts” that “we are born with all the eggs we will ever have” and “the quantity and quality of eggs declines with age,” these statements convey a temporal logic in which fertility is continually slipping away – a slippage that may be halted with OC: “freezing eggs is like freezing time” (Kindbody 2018a). In keeping with this logic, OVA Egg Freezing states:

Your fertility is never going to be as young as it is today–so why wait? (2017)

This emphasis on the ongoingness of fertility decline – and the suggested urgency of freezing eggs as early as possible – coincides with a push to market egg freezing to younger women. Prelude Fertility’s president Susan Herzberg, for example, states that egg freezing “used to resonate primarily with women in their late 30s,” but Prelude is “now targeting women in their 20s and early 30s” (Ferla 2018). The senior OVA nurse specialist and “Bachelor” reality TV winner Whitney Bischoff likewise asserts that “we really want to [reach] the younger crowd because that’s the best time to do it” (2015).^5^

This trend is coupled with fertility financing plans that enable this approach to early freezing. As Emily Jackson writes,

the representation of egg freezing as a responsible choice for all women who might want to have children in the future is at odds with […] its unaffordability for almost all women. (2017, 30)

Because younger women especially are less likely to be able to afford OC – costs average around $10.000/cycle – these marketing efforts are often paired with payment plans. Within a treatment rationale that promotes earlier freezing, it is better to freeze young eggs now and pay later, rather than save up and freeze older eggs. In this way, the capital investments in the promise of the expansion of egg freezing as a mainstream practice are complemented with additional revenue produced through financial instruments such as fertility loans and subscription plans. Consequently, broadening the target group for egg freezing can increase revenue by creating new norms and needs for both clinical and financial services.

The distribution of consumer credit through clinics is widespread throughout the fertility industry. The average cost for an IVF cycle in the US was $13.048 and a recent survey showed almost all infertility physicians identified cost as the largest barrier to care (McLaughlin *et al*. 2018). Almost 50% of US fertility clinics mention credit on their websites, often through third-party fertility lenders, such as CapexMD, IntegraMed and Prosper (Hawkins 2009, 863; Jacoby 2009, 148). Reflecting a national context characterized by a fee-for-service healthcare and higher treatment fees, 70% of women using fertility treatment in the US accrued debt. Almost half of these women incurred over $10.000 in debt and younger women (25-34) borrowed significantly more than their seniors (Market Cube 2015). Firms in the industry estimate fertility-related loans totaled about $4 billion in 2011 (Silver-Greenberg 2012).

Although the debt financing of IVF can expand access, legal scholars have raised concerns about the potential conflict of interest arising from arrangements between clinics and lenders, given the power and trust relation between doctors and patients and the potential financial incentives for prescribing both particular treatments and the means to finance them (Jacoby 2009; Hawkins 2009). So while they may be valuable to patients struggling to afford treatment, fertility loans may also change the dynamics between financial and reproductive decision making for patients and professionals alike. Nonetheless, as Melissa Jacoby asserts, fertility companies that wish to expand “must move beyond the elite to those of more-modest means. Specialty consumer credit could be a key ingredient to this expansion, particularly when partnered with other financial products” (2009, 170, 175).

**Figure 3:**
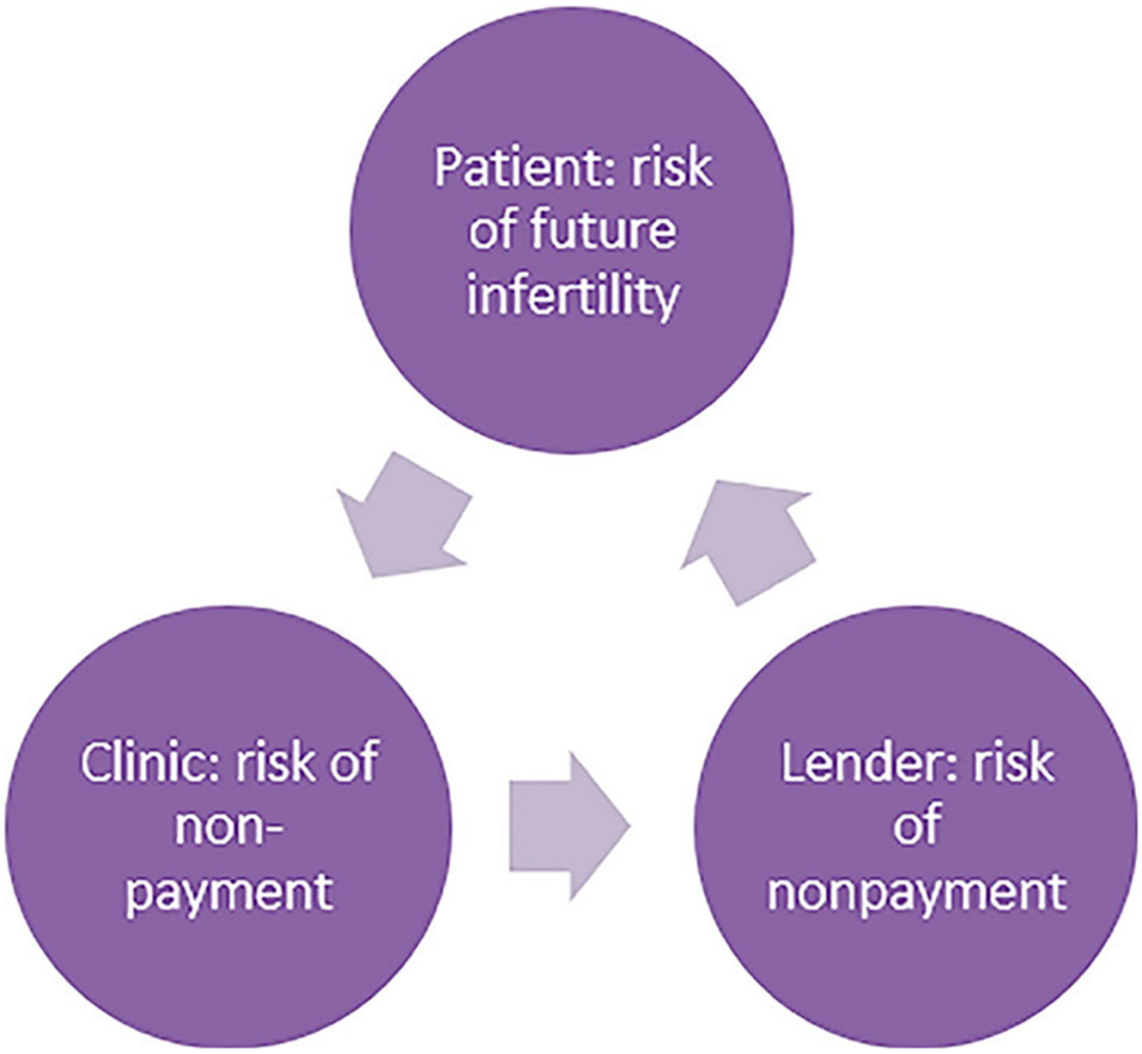
Debt financing model of egg freezing.

Similarly, the egg freezing companies’ encouragement of earlier freezing may also entail an invitation into a debt relation between patient and the fertility (financing) company. The creation of dedicated fertility lending companies attests to the fact that the debt financing of egg freezing functions as a revenue source in its own right. Companies such as Extend Fertility work with external lenders for their subscription plans, which charge between 7% and 22% interest rates and 1%–6% origination fees, depending on one’s credit score. In this way, the financial risk taken by clinics to recruit younger people with less financial means is transferred to lenders, who subsequently pass this risk on to patients through varying rates and fees – in line with Lazzarato’s observation that financialized capitalism demands “that one take upon oneself the costs and risks externalized by […] corporations” (2012, 51). In this process, value is created through a circular shifting of financial and reproductive risk: as patients shift the risk of future infertility to the clinic, clinics transfer the risk of nonpayment to lenders, who, in turn, move this risk to patients through differential rates and fees (Figure 3). In this dynamic exchange of reproductive and financial risk, fertility lending thus aligns companies’ capital accumulation with patients’ fertility accumulation through OC. By promoting both a proactive treatment rationale and fertility financing, this *debt financing model of egg freezing* creates value through a double temporal movement of anticipation and deferral; it combines treating future infertility in the present and paying for present treatment in the future.

Lastly, besides fertility financing, fertility insurance is another financial product that is rapidly growing in popularity as a result of capital investments in cryopreservation. Having secured almost $100M in venture capital, market leader Progyny has a widespread reach with its online platforms, over 500 affiliated clinics and coverage of a purported 1 million people (CNBC 2018; Progyny 2019). Its fertility benefit streamlines egg freezing into an elaborate IVF package presented as “the first end-to-end proactive fertility solution for both large, self-insured employers [and] today’s informed consumer looking to manage their reproductive health” (Progyny 2016). Progyny presents its proactive fertility program to employers as a means to improve return on investment (ROI) both by limiting costs for absenteeism and multiple pregnancies associated with “reactive” IVF and by fostering a “family friendly” and innovative image (Abdou 2016). Progyny thus integrates proactive fertility management into the workplace by positioning OC as a tool for employees to self-invest in future fertility and a tool for employers to increase ROI. Significantly, by aligning the financial investment rationales for employers and the reproductive investment rationales for their patient-employees, Progyny institutionalizes a speculative approach to fertility, which positions egg freezing as the entry point into a long-term, highly-technologized, proactive fertility management plan for a growing number of women.

## Conclusion

This article focuses on the remarkable emergence of egg freezing in the last decade and explores the ways in which processes of financialization play a central role in the organization of contemporary US IVF – and the widespread mainstreaming of OC in particular. Situated in a broader context of financialized capitalism, the growing popularity of egg freezing is propelled by large capital investments in cryopreservation in recent years. The growth potential of egg freezing as a widely-indicated treatment and the promissory nature of proactive fertility preservation align directly with the logic of “promissory capitalism” underlying equity investment markets (Hogarth 2017, 266). It is therefore not surprising that the niche of (oocyte) cryopreservation has been particularly successful in attracting finance capital and, consequently, egg freezing is now at the heart of a consolidating trend of the US fertility industry that is both reorganizing the sector and changing the discursive construction of fertility through these growing enterprises.

As became clear in the case of Prelude Fertility, equity-backed expansion, acquisition and consolidation strategies can subsume traditional IVF practices under the umbrella of growing egg freezing enterprises. Even when clinics are not directly acquired, the egg freezing companies have a widespread reach through marketing efforts directed at broader target groups and financial products that cover treatment costs by bundling egg freezing with other treatments. By bringing together payment, telemedicine care and fertility information, centralized online platforms moreover become key framing instruments for organizing and promoting egg freezing treatment across nation-wide networks.

The major egg freezing companies also offer financial products such as subscription and insurance plans. Subscription plans are presented as a means to democratize access to treatment, yet, in doing so, they set up a dynamic of investment and indebtedness in the process of preserving fertility. Characteristic of financialization, this brings debt relations to the heart of assisted reproduction and sets up additional sources of OC-related revenue through financial instruments, while enabling more spending on treatment cycles. Fertility insurance displaces the promissory value and speculative investment associated with egg freezing to the level of the employer and thereby integrates the (financial) management of fertility into the realm of labor. Both subscription and insurance products streamline egg freezing into a wider set of treatments, thereby adopting OC as a stepping stone into a longer-term trajectory of proactive technologized fertility management.

By means of the expansive growth and reach of fertility companies – through mergers and acquisitions, network formation, online marketing and financial products – egg freezing is thus changing the landscape of IVF. The financialization of fertility, in this context, references the significant financial investments in a future in which ever more women freeze their eggs, the role of private equity and venture capital in establishing the clinical and commercial infrastructures through which egg freezing becomes accessible, the alignment of the financialized logics of the capital market and those underlying dominant treatment rationales and the role of financial products in shaping both the stories and the streamlining of fertility preservation. Together, these developments are indicative of a shift from reproduction to fertility in IVF, in which treatment need not necessarily be aimed at having a child in the face of infertility, but rather at the proactive management of a more speculative fertility throughout the life course. As a result, the introduction and financial backing of egg freezing presents not simply another reproductive option, but has instigated a step-change in IVF and is changing what it means to be fertile in the twenty-first century.

## Notes

1 This study focuses specifically on the new equity-backed start-up companies specialized in egg freezing, rather than IVF clinics in general. Information about the amount of equity funding received by these companies is in the public domain and may be accessed through online databases such as Crunchbase. The paper draws on Vertommen’s genealogical method for analyzing the “reproductive-industrial complex” as well as Bal’s method of cultural analysis by reading key discursive objects that organize the marketization of egg freezing – including online platforms, press releases and annual reports – in relation to a body of scholarship in reproductive studies and the political-economy of biotechnology (Vertommen 2017; Bal 2002).

2 In keeping with the article’s focus, I read egg freezing in relation to financialized capitalism. The emergence of egg freezing technology and practice is not reducible to this context, but the result of a complex history starting with the birth of the first frozen-egg baby in 1986 and developed with the aid of private and public funding, in relation to various indications (oncological, age-related, ethical) and in a variety of regulatory climates, which may spur on egg freezing in response to restrictions on the use of embryos or incentives for a new commercially-viable treatment for reproductive ageing (Chen 1986). For a more detailed discussion, see Martin (2010), Waldby (2019) and Van de Wiel (forthcoming).

3 Estimations of the IVF market size vary wildly and methods for determining these figures are not transparent. What is clear across different IVF market reports, however, is the ongoing growth and expansion of the sector.

4 These fertility benefits are particularly popular in the tech industry. Companies who confirmed they offer egg freezing benefits to female staff and employees’ spouses include Facebook, Apple, Google, Uber, Yahoo, Netflix, Snapchat, Intel, eBay, Time Warner, Salesforce, LinkedIn and Spotify (Kerr 2017). Egg freezing is covered by 10% of US employers with 20.000 or more employees (Mercer 2016).

5 Commenting on US egg freezing more broadly, the ASRM ethics committee notes that it is “concerned about […] the line between education of young women and inappropriately aggressive marketing to them.” They recommend that providers disclose clinic-specific statistics as well as the fact that medical benefits and harms of OC are not yet fully understood (ASRM 2018). In their analysis of the quality of information about social egg freezing on the websites of 147 US clinics, Avraham and colleagues found that the majority of websites did not follow the ASRM guidelines on OC and related advertising (2014).
